# The immunomodulatory functions and molecular mechanism of a new bursal heptapeptide (BP7) in immune responses and immature B cells

**DOI:** 10.1186/s13567-019-0682-7

**Published:** 2019-09-18

**Authors:** Xiu Li Feng, Yang Zheng, Man Man Zong, Shan Shan Hao, Guang Fang Zhou, Rui Bing Cao, Pu Yan Chen, Tao Qing Liu

**Affiliations:** 1 Key Laboratory of Animal Microbiology of China’s Ministry of Agriculture, College of Veterinary Medicine, Nanjing Agricultural University, Nanjing, 210095 China; 2 MOE Joint International Research Laboratory of Animal Health and Food Safety, College of Veterinary Medicine, Nanjing Agricultural University, Nanjing, 210095 China; 3 Institute of Veterinary Medicine, Jiangsu Academy of Agricultural Sciences, Nanjing, 210014 China

## Abstract

The bursa of Fabricius (BF) is the acknowledged central humoural immune organ unique to birds and plays a vital role in B lymphocyte development. In addition, the unique molecular immune features of bursal-derived biological peptides involved in B cell development are rarely reported. In this paper, a novel bursal heptapeptide (BP7) with the sequence GGCDGAA was isolated from the BF and was shown to enhance the monoclonal antibody production of a hybridoma. A mouse immunization experiment showed that mice immunized with an AIV antigen and BP7 produced strong antibody responses and cell-mediated immune responses. Additionally, BP7 stimulated increased mRNA levels of sIgM in immature mouse WEHI-231 B cells. Gene microarray results confirmed that BP7 regulated 2465 differentially expressed genes in BP7-treated WEHI-231 cells and induced 13 signalling pathways and various immune-related functional processes. Furthermore, we found that BP7 stimulated WEHI-231 cell autophagy and AMPK-ULK1 phosphorylation and regulated Bcl-2 protein expression. Finally, chicken immunization showed that BP7 enhanced the potential antibody and cytokine responses to the AIV antigen. These results suggested that BP7 might be an active biological factor that functions as a potential immunopotentiator, which provided some novel insights into the molecular mechanisms of the effects of bursal peptides on immune functions and B cell differentiation.

## Introduction

Undoubtedly, the most significant contribution that studies on the avian immune system have made to the development of mainstream immunology has been delineating the two major arms of the adaptive immune system, namely, humoural and cellular immunity [1–4]. Since surgical removal of the bursa from neonatal chicks impairs subsequent antibody responses to *Salmonella typhimurium* type O antigen [1], it is clear that the BF is the key location of B cell lymphopoiesis in birds [3, 4].

B cell development occurs in three distinct stages, namely, pre-bursal, bursal and post-bursal stages, and each of these stages plays a fundamentally different role in B cell development [5]. Furthermore, Liu et al. [6] reported the transcriptional changes in mRNA expression in different developmental stages in the BF. A complete understanding of the anatomy and function of the BF is lacking, and the mechanism underlying the involvement of the BF in B cell development still needs to be profoundly elucidated.

B cell differentiation and antibody diversification are accompanied by the regulation of biologically active molecules and activation of immune induction [4]. Bursin tripeptide (Lys-His-Gly-NH2) was reported to be the first B cell-differentiating hormone derived from the BF [7, 8], to selectively induce avian B cell differentiation, and to promote immunoglobulin (Ig) class switching from IgM to IgG [9]. BP8, which has the sequence AGHTKKAP, can regulate various signalling pathways and retinol-binding protein expression, which represents an important link between B cell development and retinol metabolism [10]. Bursal pentapeptide (BPP)-II regulates the expression of various genes involved in homologous recombination in DT40 avian pre-B lymphocyte cells and enhances antibody production in response to chicken immunization [11]. Furthermore, BP8 can promote colony-forming pre-B cell formation and regulate B cell development [12], and BP5, with the sequence CKDVY, regulates B cell development by promoting antioxidant defence [13]. BPP-II regulates more than one thousand differentially expressed genes that are involved in various pathways and immune-related biological processes in hybridoma cells, which secrete monoclonal antibodies [14]. The avian immune system may provide important insights into fundamental immunological mechanisms, and the chicken may be the best-studied non-mammalian species [15].

To investigate the function and molecular basis of bursal-derived peptides in the immune response and immature B cells, in this study, we isolated a new peptide, BP7, from the BF with RP-HPLC and MS/MS analysis and showed the inducing roles of BP7 in immune responses to vaccination. Furthermore, we applied a gene microarray to screen the gene expression profiles of immature mouse B cells after BP7 treatment and analysed the enriched pathways and function categorization of the differentially expressed genes in the immature B cells. The results provided some vital information on the mechanisms involving the bursal peptide in immune induction and immature B cell development.

## Materials and methods

### Animal

BALB/c female mice (approximately 19 g) were obtained from the experimental animal centre of Yangzhou University (Yangzhou, China). Seventy-five-day-old female chickens were purchased from Qinglongshan Farm (Nanjing, China). Experiments were conducted following the guidelines of the Animal Ethics Committee at Nanjing Agricultural University, China. The euthanasia and sampling procedures complied with the “Guidelines on Ethical Treatment of Experimental Animals” (2006) No. 398 published by the Ministry of Science and Technology, China and “the Regulation regarding the Management and Treatment of Experimental Animals” (2008) No. 45 published by the Jiangsu Provincial People’s Government.

### RP-HPLC and MS analysis

As described previously [14] with some modifications, an extract of the BF was sequentially separated and purified to isolate soluble peptides from bursal samples. Briefly, 50 g bursa tissues from a 1-month-old AA broiler chick without fascia or adipose tissue were washed once with 0.85% physiological saline (pre-cooled to 4–10 °C) and placed in a tissue homogenizer, and then the pre-cooled 0.85% saline buffer was added to the tissues. After the homogenization process and ultrasonic treatment were completed, a pyrolysis solution was heated to 80 °C for 5 min, quickly placed on ice and cooled to 10 °C, and then centrifuged at 4000 *g*/min for 30 min. The supernatant was collected, frozen and thawed twice, and centrifuged at 12 000 *g*/min for 30 min at 4 °C. The supernatant was ultrafiltered with a molecular weight cutoff of 1000 Da and purified on a SinoChrom ODS-BP RP-HPLC affinity column (C-18 Aqua column, 150 × 4.6 mm) following a linear gradient of acetonitrile (2–100%) in Agilent 1100 series high-performance liquid chromatograph at 25 °C. The acetonitrile-soluble fraction based on the elution time was collected and analysed using a mass spectrometer (SHIMADZU LC-AB SCIEX Triple TOF 4600) and further analysed with MS/MS to obtain the exact amino acid sequence analysed by Nanjing GenScript Bioscience Co., Ltd. (Nanjing, China); the detailed information is listed in Additional file 1A.

### Peptide synthesis

A bursal-derived peptide was synthesized by Nanjing GenScript Bioscience Co., Ltd. (Nanjing, China) with a purity over 95% and was analysed by HPLC and mass spectrometry.

### Hybridoma cell treatment

Hybridoma cells (10^5^ cells/mL) were seeded in a 96-well plate and treated with or without the synthesized BP7 peptide (1, 0.1, or 0.01 μg/mL), and the antibody levels in the cell supernatant at 48 h were determined by ELISA [14, 16]. Cell viabilities were determined with an MTT reagent (Sigma) according to the manufacturer’s instructions as follows: the relative survival or antibody stimulation index (%) = ((absorbance of BP7 treatment-blank)/(absorbance of control-blank)) × 100%. Additionally, hybridoma cells were treated with BSA at 0.1 μg/mL as a control.

### Mouse immunization and detection protocols

To investigate whether BP7 performs immunoregulatory functions as an immunopotentiator, we intraperitoneally immunized mice with 0.2 mL AIV antigen with or without the synthesized BP7 peptide on days 0 and 14 according to a previously reported immune programme [14, 17]. On the 28^th^ day after immunization, we collected serum samples from all mice and detected antibody titres and antibody subtypes by ELISA [15]. On the 7^th^ day after the second immunization, serum samples were collected from all immunized mice to detect IL-4 and IFN-γ cytokine production by ELISA kits (RD, USA), and splenic lymphocytes were isolated to classify T cell immunophenotyping by using fluorophore-labelled anti-CD3, anti-CD4, and anti-CD8 antibodies. Furthermore, the splenic lymphocytes collected from all experimental groups were treated with an inactivated AIV H9N2 antigen to assess lymphocyte viabilities.

### WEHI-231 cell treatment

WEHI-231 cells were treated with the synthesized BP7 peptide at concentrations ranging from 0.01 to 1 μg/mL for 4 h, cell viability was tested with MTT methods, and the sIgM levels of WEHI-231 cells were detected with qPCR. Additionally, WEHI-231 cells were treated with 0.1 μg/mL BSA as a control. The primers for mouse sIgM were primer-F-gtggaatctggcttcaccac and primer-R-cattcaggttcagccagtcg.

### Microarray assay and data analysis

According to previous methods [14, 18] with some modifications, total RNA was extracted from WEHI-231 cells treated with 0.1 μg/mL BP7 for 4 h by using TRIzol reagent (Invitrogen) and processed for hybridization according to the manufacturer’s instructions. Three independent experiments were performed. Gene expression profiles were detected with an Agilent mouse (V2) gene expression microarray (8 × 60 K, Agilent) and scanned with the Agilent G2565CA Microarray Scanner. The resulting data were analysed with AgilentGeneSpring GX software. A fold change of 2.0 with a 95% significance level was selected as the threshold for comparisons between paired cell lines. For the exploration of pathways and processes involved in the development of immature B cells in WEHI-231 cells, we also conducted a functional analysis with a threshold of a *p* value less than 0.05.

### Quantitative real-time reverse transcription PCR

Total RNA was prepared from WEHI-231 cells treated with 0.1 μg/mL BP7 using TRIzol reagent. Six significantly differentially expressed genes in WEHI-231 cells were checked using the One Step SYBR^®^ PrimeScript™ RT-PCR Kit (Takara, Shiga, Japan), including Sos1, Atg14, Atg12, Ube2b, Pias3, and Ifnb1. qRT-PCR was performed using an ABI 7300 Real-Time PCR system (Applied Biosystems) according to the manufacturer’s specifications. The primers for these selected genes in WEHI-231 cells, which were analysed with the 2^−ΔΔCt^ method using Actin as an internal control, are shown in Additional file 2.

### Transmission electron microscopy

According to reported methods [19], WEHI-231 cells were treated with 10 μg/mL synthesized BP7 for 24 h and then collected by centrifugation at 1000 rpm for 10 min. BSA (10 μg/mL) was used as a control. After washing, the WEHI-231 cells were fixed with 2.5% glutaraldehyde and 1% osmium tetroxide. After dehydration with a 50–100% (with a 10% gradient) series of ethanol and pure acetone, the WEHI-231 cells were embedded, cut and stained with uranyl acetate and lead citrate Zi to image the autophagosomes in the WEHI-231 cells with a H7650 transmission electron microscope (HITACHI, Japan).

### Western blot analysis

WEHI-231 cells were treated with the synthesized BP7 peptide at concentrations ranging from 0.001 to 50 μg/mL for 24 h, and then total protein was isolated from the WEHI-231 cells using a cell culture lysis reagent (Promega) following the manufacturer’s instructions. Western blotting was performed as described previously [19] using mouse anti-mouse monoclonal antibodies against LC3 (E1A4007-1, EnoGene, China), AMPK (5832T, CST), p-AMPK (2535T, CST), ULK1 (8054T, CST), p-ULK1 (5869T, CST), and Bcl-2 (E1A6139, EnoGene, China). The expression of the internal reference protein GAPDH was detected with a rabbit anti-mouse GAPDH antibody (E12-052-1, EnoGene). Additionally, WEHI-231 cells were treated with 10 μg/mL BSA as a negative control or rapamycin as a positive control.

### Avian immunization

Seventy-five-day-old chickens were subcutaneously injected twice with 0.2 mL AIV vaccine with or without 0.05 mg/mL synthesized BP7 on days 0 and 14. On the 14^th^ and 28^th^ day after immunization, we collected serum samples from all immunized chickens to detect HI antibody titres. Additionally, on the 7^th^ day after the second immunization, serum samples were collected to detect IL-4 and IFN-γ cytokine production by ELISA kits (RD), and splenic lymphocytes were isolated to detect lymphocyte viability.

### Statistical analysis

The results are illustrated in bar graphs as the mean ± standard deviation (SD) of three independent experiments. Statistical significance was analysed by *t* tests or one-way ANOVA with the threshold for a significant difference set at 0.05.

## Results

### Isolation and identification of the bursal-derived peptide BP7

**Figure 1:**
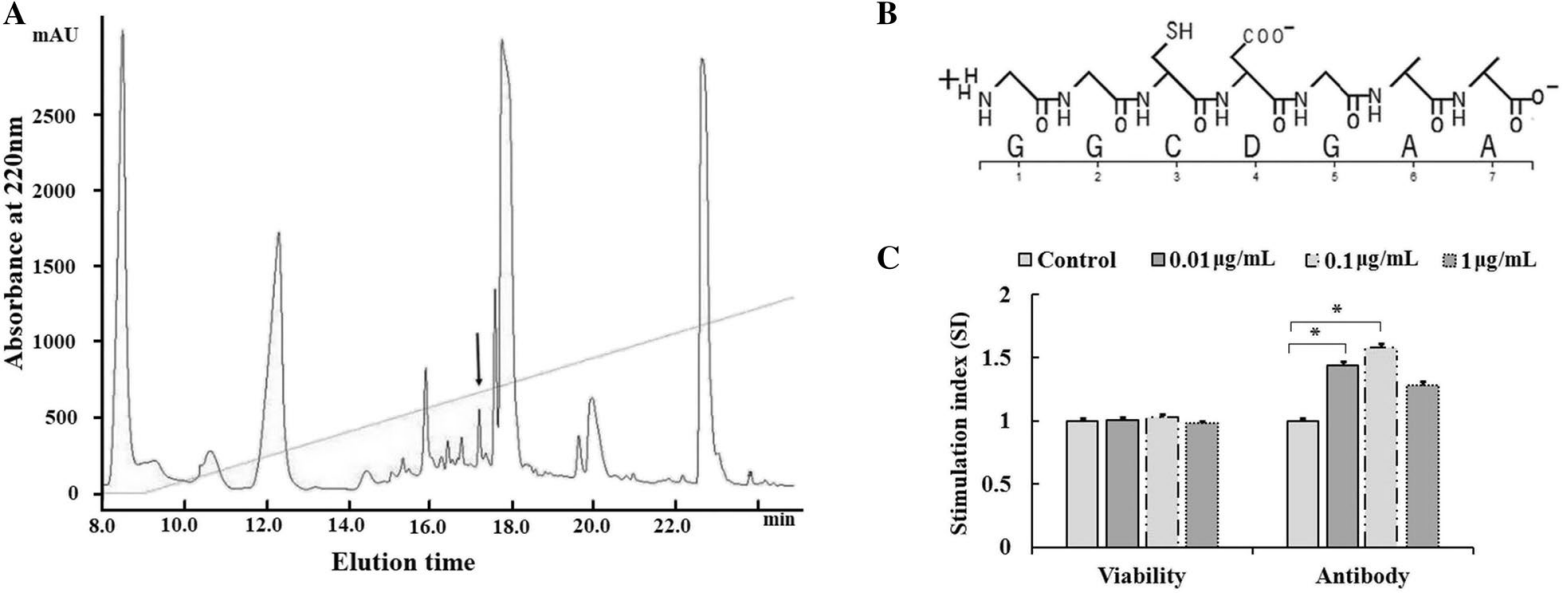
**Isolation and identification of BP7. A** The isolation and purification of BP7. **B** MS/MS analysis of the amino acid sequence of BP7. **C** Antibody production of hybridoma cells enhanced by BP7. Data represent the mean ± standard deviation (s.d.). Significant differences between groups were determined using Student’s *t*-test, and *p* < 0.05 is indicated by an asterisk (*).

In this paper, an ultrasonic sample of a bursal extract was isolated and analysed by RP-HPLC coupled to MALDI-TOF-MS and MS/MS analysis, and a new bursal peptide was separated in a peak at 17.09 min (Figure 1A). The amino acid sequence was analysed with MS/MS (Additional file 1B), a seven-amino acid sequence, GGCDGAA (namely, BP7), was obtained (Additional file 1C), and the chemical structure is shown in Figure 1B.

By aligning the sequence of BP7 in the non-redundant and Expressed Sequence Tag databases of NCBI, we found that BP7 was similar to proteins from *Gallus gallus* and mice, suggesting that BP7 was conserved in both these species and was probably a proteolytic degradation fragment of an intact protein; there were two proteins of interest in *G. gallus* (Additional file 3) including interferon-induced helicase C domain-containing protein 1 (IFIH1; NP_001180567.1) and immunoglobulin heavy chain variable region (IGHV; CAO79246.1). Additionally, we blasted the amino acid sequence GGCDGAA in *Lactobacillus* and *Escherichia coli* and did not find proteins in these bacteria with exactly the same sequence as that of BP7.

To confirm the inducing role of BP7 in antibody production, hybridoma cells secreting an antibody specific for JEV were used as an in vitro model [16]. The results showed that compared to control treatment, BP7 treatment enhanced the monoclonal antibody production levels of the hybridoma cells by ELISA (Figure 1C), in which the antibody levels were increased by 45.91%, 52.1% and 27.55% at 0.01, 0.1 and 1 μg/mL BP7 treatment, respectively. However, hybridoma cell proliferation did not significantly differ with 0.01 to 1 μg/mL BP7 treatment compared with control treatment (Figure 1C).

### BP7 induced an immunomodulatory response to immunization in mice

**Figure 2:**
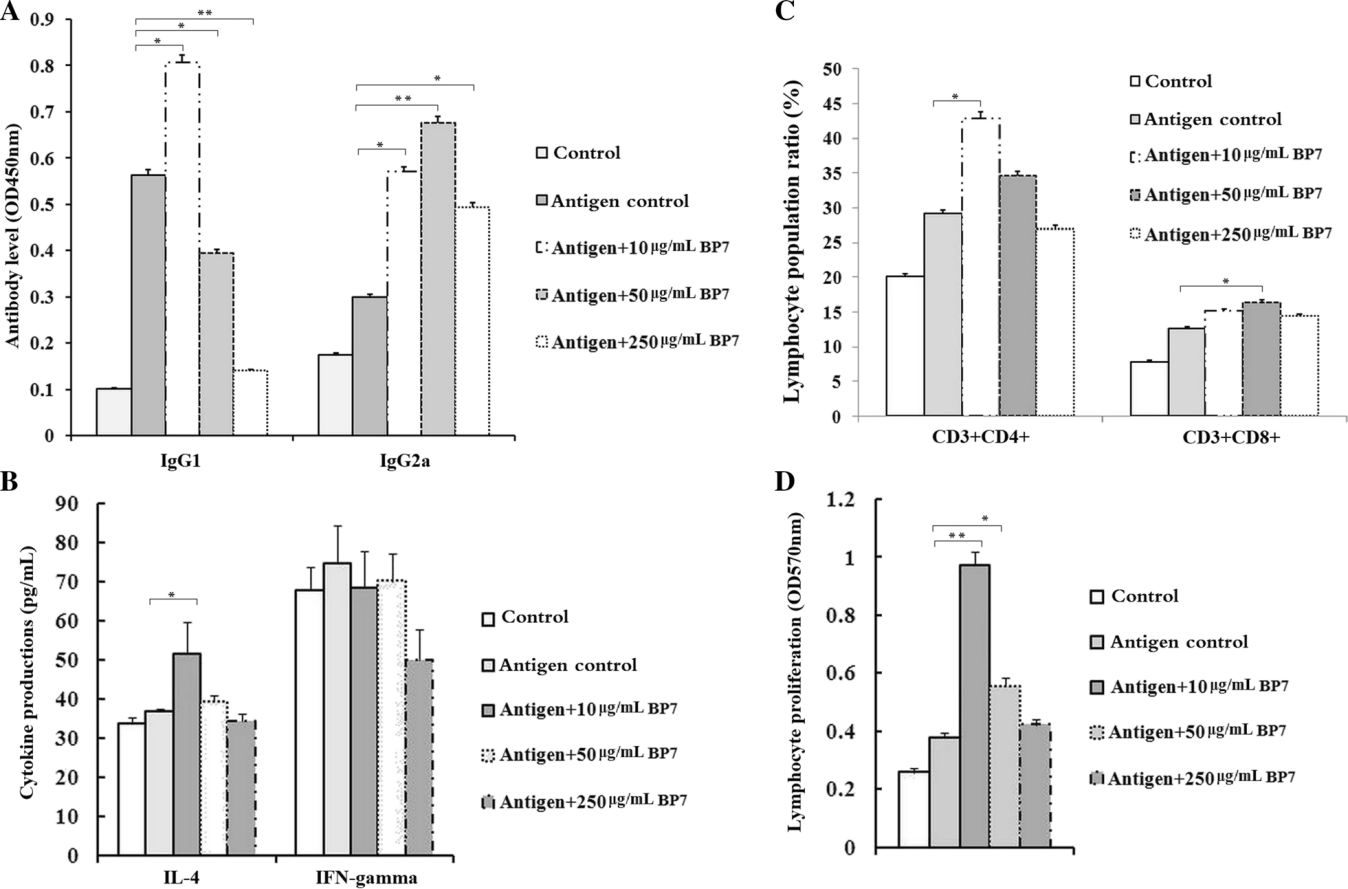
**BP7 induced various immune responses in mice immunized with an AIV antigen.** BALB/c mice were immunized with BP7 and an AIV H_9_N_2_ antigen following a prime-boost vaccination strategy. **A** Antibody subtypes, **B** cytokine production, **C** T cell populations, and **D** lymphocyte viability. Data represent the mean ± standard deviation (s.d.). Significant differences between groups were determined using Student’s *t*-test, where *p* < 0.05 and *p* < 0.01 are indicated by asterisks (* and **, respectively).

To evaluate the immune-inducing roles of BP7 in immunization, the subtypes of AIV-specific antibodies present at 4 weeks after immunization were analysed by ELISA. For IgG1, all serum samples were diluted 1:10^4^. It was observed that mice immunized with an AIV antigen and three concentrations of BP7 produced significantly increased IgG1 antibody levels compared with mice immunized with a control antigen, and the IgG1 levels of the mice immunized with the AIV antigen and 50 μg/mL BP7 were the highest among those of the mice in all experimental groups (Figure 2A). Additionally, the serum samples from the mice in all groups were diluted at 1:10^3^ to detect IgG2a levels. We found that the mice immunized with the AIV vaccine and BP7 produced higher levels of IgG2a antibodies than the mice immunized with the control antigen, and the elevated levels exhibited a dose-dependent pattern (Figure 2A).

To assess the capability of BP7 to elicit Th1- and Th2-type immune responses, on the 7^th^ day after the second immunization, we detected the levels of the cytokines IFN-γ and IL-4 in serum samples from all groups using a mouse ELISA kit. The data suggested that the production of IL-4 in the mice immunized with the AIV antigen and 10 μg/mL BP7 was significantly higher than that in those immunized with a control AIV antigen, while the levels of IFN-γ were not significantly different among all immunization groups, although decreased IFN-γ production was observed in the mice treated with the AIV antigen and 250 μg/mL BP7 (Figure 2B).

Additionally, 1 week after the second immunization, CD4+ T cell percentages in the mice immunized with 10 or 50 μg/mL BP7 were significantly higher than those in the control mice, and CD8+ T cell percentages in the mice immunized with 50 μg/mL BP7 were significantly higher than those in the control mice (Figure 2C). After antigen stimulation, the cell viabilities in the mice immunized with the AIV antigen and 10 or 50 μg/mL BP7 were significantly higher than those in the control mice (Figure 2D).

### Inducing effects of BP7 on immature B cells

**Figure 3:**
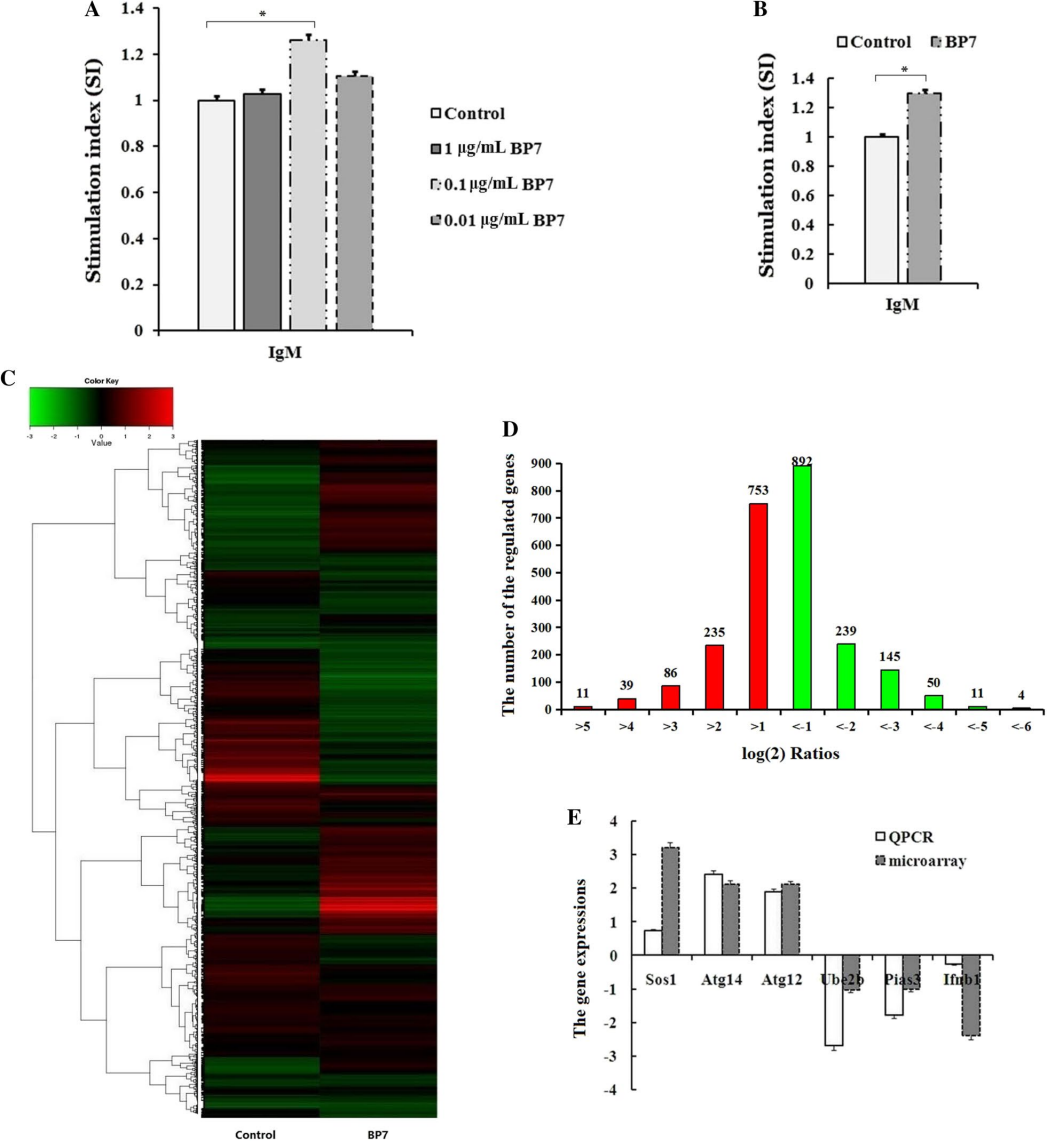
**Roles of BP7 and BP7-induced gene expression profiles in WEHI-231 cells.** WEHI-231 cells were treated with BP7 for 4 h. sIgM levels were detected by qPCR (**A**, **B**). **C** A heat map shows the gene expression profiles of BP7-treated WEHI-231 cells. **D** The histogram presents differentially expressed genes. **E** The differentially expressed genes in WEHI-231 cells were validated. Data represent the mean ± standard deviation (s.d.). Significant differences between groups were determined using Student’s *t*-test, where *p* < 0.05 is indicated by an asterisk (*).

To detect the roles of BP7 in immature B cells, WEHI-231 cells were used as a mouse model of immature B cells and treated with BP7 at concentrations ranging from 0.01 to 1 μg/mL. As shown in Figure 3A, the sIgM level in the WEHI-231 cells treated with 0.1 μg/mL BP7 was significantly higher than that in the PBS-treated control cells. Additionally, the sIgM level in the WEHI-231 cells treated with 0.1 μg/mL BP7 was higher than that in the BSA-treated control cells (Figure 3B).

### Gene expression profile analysis of WEHI-231 cells that received BP7 treatment and validation

To investigate the gene expression patterns induced by the bursal-derived peptide in immature B cells as well as the associated functional classifications, mouse WEHI-231 cells were treated with 0.1 μg/mL BP7 for 4 h, and the gene expression profiles of the BP7-treated WEHI-231 cells were analysed by a mouse cDNA microarray. Heat maps illustrate the overall expression profiles of the WEHI-231 cells treated with BP7 (Figure 3C). With the use of specified thresholds, 1124 upregulated genes and 1341 downregulated genes were observed in the BP7-treated WEHI-231 cells compared with the control cells (Figure 3D, Additional file 4); the fold changes in the expression of most genes in the WEHI-231 cells and DT40 cells given BP7 treatment were between 1 and − 1 log (2) ratios (Figure 3D).

To validate the differentially expressed genes by gene microarrays, the expression levels of six genes were determined using qRT-PCR. The results showed that the expression levels of Sos1, Atg14 and Atg12 were higher and those of Ube2b, Pias3 and Ifnb1 were lower in the BP7-treated WEHI-231 cells than in the control cells (Figure 3E). These results were consistent with the microarray analysis of BP7-treated WEHI-231 cells.

### BP7 induced enriched signals and pathways in WEHI-231 cells

**Figure 4:**
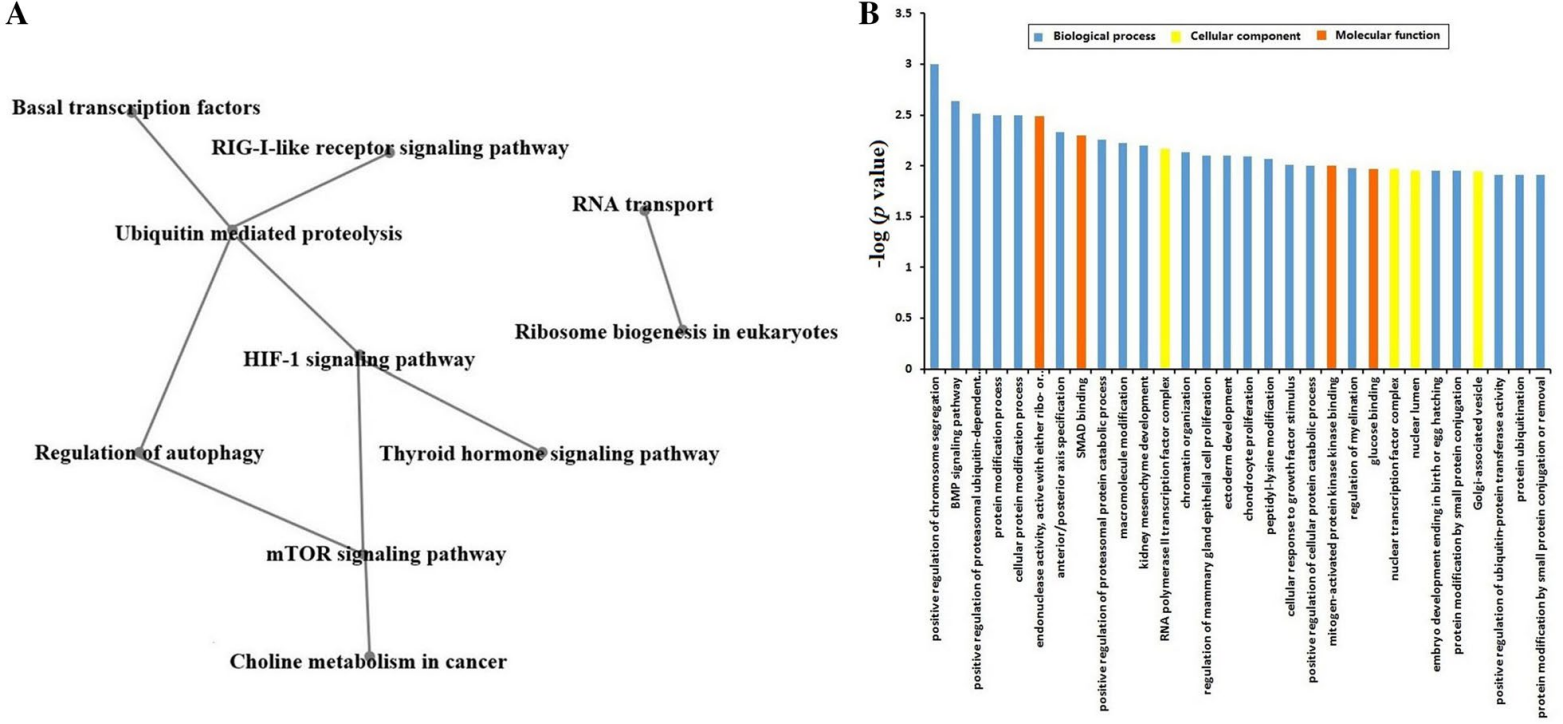
**Pathway interaction network and significant GO terms with a** ***p*** **value within TOP30 in WEHI-231 cells treated with BP7. A** The pathway network analysis of 13 significantly enriched pathways (shown in Additional file 5) and **B** summarized significant GO terms with a *p* value within TOP30.

To attain an overview of biological pathway regulation in immature B cells, we analysed signalling and metabolic pathways in the Kyoto Encyclopedia of Genes and Genomes (KEGG) database. It was found that the differentially expressed genes induced in WEHI-231 cells by BP7 were involved in 13 pathways, of which five were related to biosynthesis and metabolism, five were related to signalling pathways, and three were related to other pathways (Additional file 5). Furthermore, we analysed the enriched-pathway network of WEHI-231 cells given BP7 treatment, as shown in Figure 4A. Ubiquitin-mediated proteolysis, which was related to various pathways in the BP7-treated WEHI-231 cells, was the most enriched pathway among all the involved pathways in the WEHI-231 cells treated with BP7.

### BP7 induced various GO functional processes in WEHI-231 cells

To further investigate the molecular basis involving BP7 in the biological functions of immature B cells, the genes with differential expression in WEHI-231 cells after BP7 treatment were analysed based on GO terms, and the significantly enriched terms with *p* values within TOP30 are summarized in Figure 4B. There were 22 biological processes, four cellular components, and four molecular functions among the GO terms with *p* values within TOP30 in the BP7-treated WEHI-231 cells (Figure 4B).

### Differential expression of immune-related functional processes in BP7-treated WEHI-231 cells

To characterize the molecular signature of the host immune system, we analysed the expression of genes involved in immune-related functional processes, which included T cell proliferation and activation, antigen processing and presentation, T helper 2 cell differentiation, the MHC I and II biosynthetic process and regulation, interleukin and autophagy in the mouse B cell line WEHI-231 after BP7 treatment. The differentially expressed genes are shown in Additional file 6.

### BP7 induced autophagy and AMPK-ULK1 phosphorylation in immature B cells

**Figure 5:**
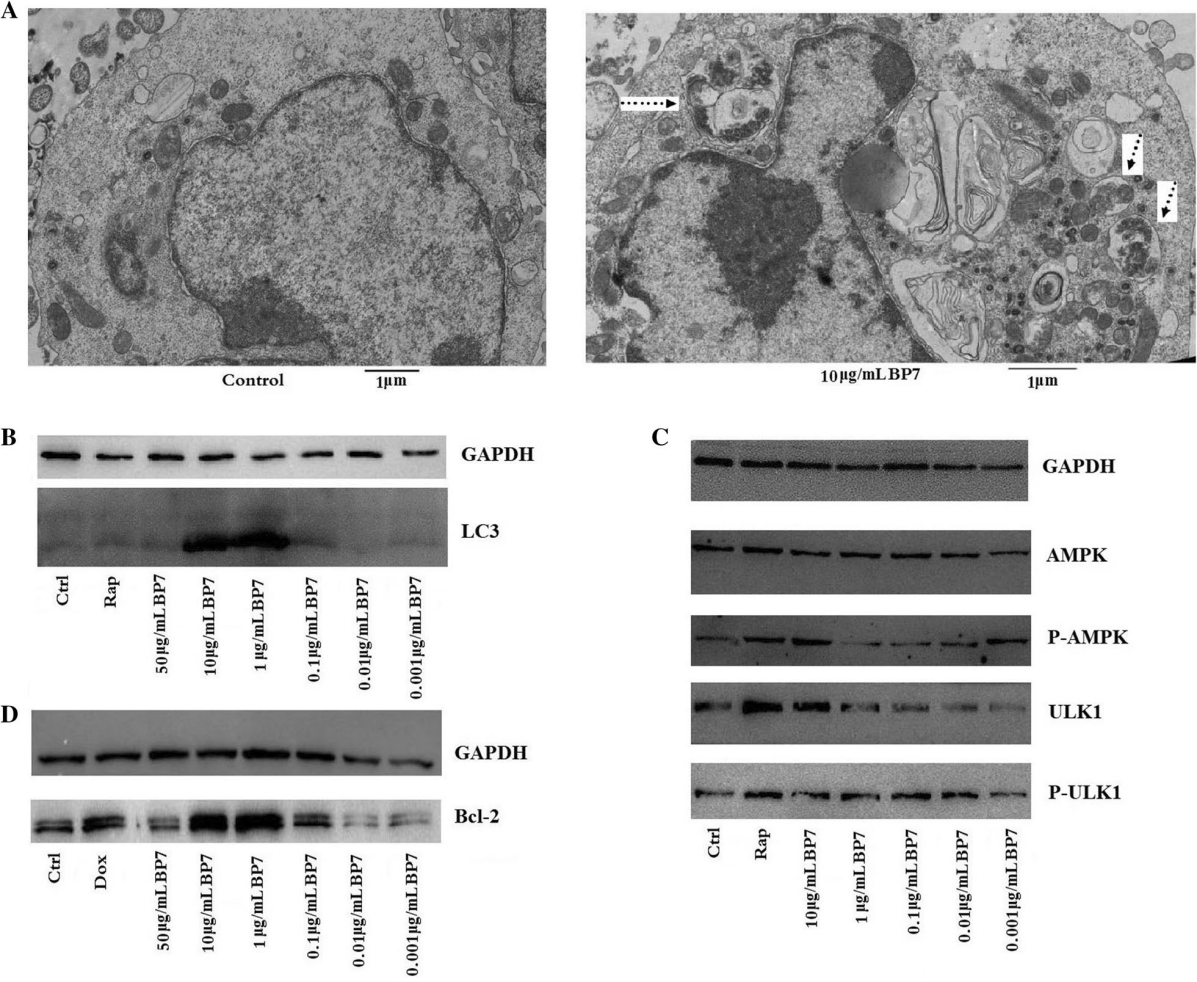
**BP7 induced autophagy and enhanced AMPK-ULK1 phosphorylation and BCL-2 expression in WEHI-231 cells. A** Transmission electron microscopy of autophagosome formation in WEHI-231 cells treated with 10 μg/mL BP7. The arrow indicates autophagosomes in WEHI-231 cells. **B** LC3 protein expression. **C** AMPK-ULK1 phosphorylation. **D** Expression levels of BCL-2. In **B** and **C**, WEHI-231 cells treated with 10 μg/mL BSA were used as the irrelevant control (Control), and those treated with rapamycin were used as the positive control. In **D**, WEHI-231 cells treated with 10 μg/mL BSA were used as the irrelevant control (Control), and those treated with Dox were used as the positive control. Representative Western blots are shown.

To detect the role of BP7 in autophagy in WEHI-231 cells, autophagosome formation was observed using a transmission electron microscope. The results showed that compared with control cells, WEHI-231 cells treated with 10 μg/mL BP7 exhibited significant autophagosomes (Figure 5A). Additionally, the protein levels of LC3 at experimental concentrations of 10 and 1 μg/mL were increased in BP7-treated WEHI-231 cells (Figure 5B). These results indicated that BP7 could induce autophagy in immature B cells.

To investigate the potential signalling involving BP7 in autophagy in immature B cells, in this paper, we detected AMPK-ULK1 phosphorylation in WEHI-231 cells treated with BP7. It was observed that 10 μg/mL BP7 stimulated the expression of p-AMPK (Figure 5C). Additionally, we observed that 10 μg/mL BP7 induced increased expression of ULK1, and BP7 at concentrations ranging from 0.01 to 10 μg/mL stimulated increased expression of p-ULK1 in treated WEHI-231 cells compared with control cells (Figure 5C). These results suggested that AMPK-ULK1 phosphorylation might be the activating signal for autophagy in immature B cells treated with BP7.

Furthermore, we found that the expression of Bcl-2 was significantly increased with BP7 treatment at concentrations of 1 and 10 μg/mL compared to control treatment (Figure 5D), and 1 μg/mL BP7 induced the highest expression of Bcl-2 among all experimental treatments.

### BP7 induced a strong immune response to immunization in chickens

**Figure 6:**
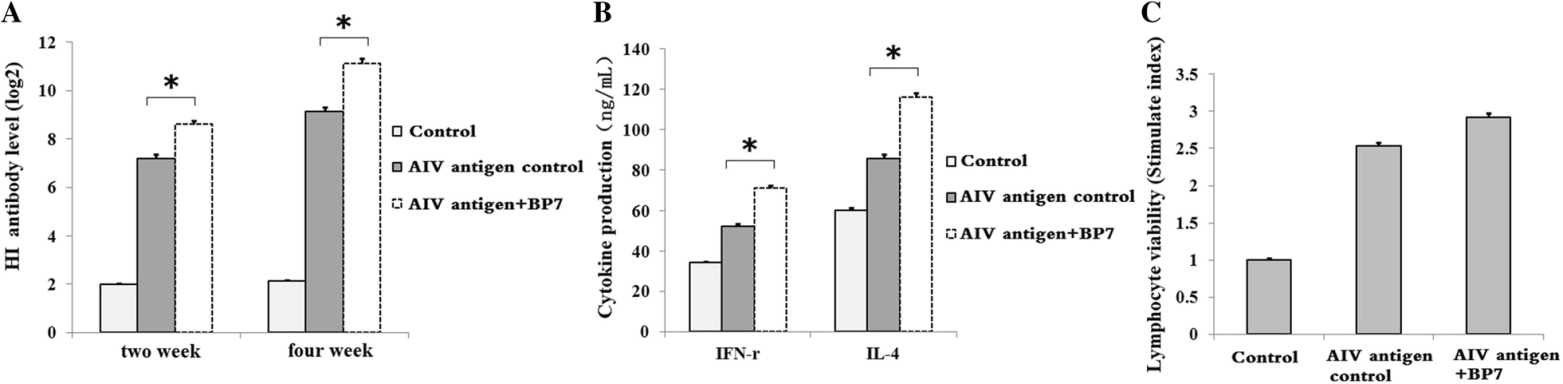
**Inducing roles of BP7 in a chicken immunization model.** Chickens were immunized twice with BP7 and an AIV H_9_N_2_ antigen. **A** HI antibody levels. **B** IL-4 and IFN-γ cytokine production. **C** Lymphocyte viability. Data represent the mean ± standard deviation (s.d.). Significant differences between groups were determined using Student’s *t*-test, where *p* < 0.05 is indicated by an asterisk (*).

To investigate the roles of BP7 in avian immunization, 75-day-old chickens were immunized twice with an AIV antigen and 50 μg/mL BP7. The results showed that at 2 and 4 weeks after immunization, HI antibody titres in the chickens immunized with the AIV antigen and BP7 were higher than those in the antigen control-immunized chickens (Figure 6A). Additionally, on the 7^th^ day after the second immunization, the levels of the cytokines IL-4 and gamma IFN in the chickens immunized with the AIV antigen and BP7 were higher than those in the antigen control-immunized chickens (Figure 6B). Additionally, we observed that the splenic lymphocyte viabilities of the chickens immunized with the AIV antigen and BP7 were higher than those of the antigen control-immunized chickens (Figure 6C). These results suggested that BP7 might induce strong immune responses in avian immunization models.

## Discussion

The BF is the central organ for B cell development in birds and is the primary site of B cell lymphopoiesis [2–4]. However, few studies have reported the unique molecular basis and mechanism of bursal-derived biological peptides in immune responses and immature B cell development.

In this paper, one new heptapeptide (BP7) was isolated from the avian bursa and found to be homologous to various proteins in *G. gallus* and *Mus musculus*, suggesting that BP7 is conserved among various species; BP7 was also found to be homologous to interferon-induced helicase C domain 1 protein (IFIH1) and immunoglobulin heavy chain variable region (IGHV) in *G. gallus*. IFIH1 can function as a pattern recognition receptor that activates innate immune responses upon binding with damage-associated molecular patterns [20, 21]. Another homologous protein, immunoglobulin heavy chain variable region (IGHV), was reported to be significantly correlated with Tfh17 cells [22] and relevant somatic mutations, such as antigen-driven affinity maturation [23]. Furthermore, BP7 stimulated increased monoclonal antibody production in hybridoma cells. Antibody production is one of the vital behaviours for B cell differentiation and maturation. These results indicated that BP7 could be an inducing factor in antibody production and B cell development.

Additionally, we performed a blast search for proteins homologous to BP7 with the amino acid sequence of GGCDGAA in *Lactobacillus* and *E. coli*, which are the bacteria commonly present in the chicken intestine, and did not find proteins in these bacteria with a sequence identical to that of BP7, which suggested that BP7 is derived from avian BF tissue. In future studies, we will further analyse the distribution and function of BP7 in the chicken BF.

Mice are a commonly used animal research model for investigating the regulatory and adjuvant functions of biologically active peptides in immune responses [14, 24, 25]. To evaluate the inducing effects on immune responses, in this paper, we primarily selected BALB/c female mice as the experimental animal model. The results proved that BP7 promoted IgG1 and IgG2a antibody production, in which IgG1 was the major antibody subtype in the mice immunized with an AIV antigen and BP7. Additionally, BP7 stimulated increases in IL-4 levels and T cell subpopulation numbers in models in immunization experiments. The IgG1 and IgG2α isotypes are both important IgG subclasses during vaccination [26, 27]. It was reported that the IgG2a subclass is a characteristic of the Th1-type immune response, while IgG1 is a representative factor of the Th2-type immune response [28, 29]. IL-4 plays an essential role during Th phenotypic development in Th2 cells, which might be important for humoural immunity and B cell development [30], and IFNγ can boost CD8+ T cell- and non-specific cell-mediated protective immune responses [29, 31]. These results suggested that BP7 primarily elicited Th1 immune responses and played a role in the Th2-type immune response in the AIV antigen-immunization model.

WEHI-231 cells are a mouse peripheral B cell model that is a common immature B cell model used to study the function and mechanism of B cells [32]. To investigate the roles of BP7 in immature B cells, we detected the roles of BP7 in WEHI-231 cells. The results proved that BP7 significantly induced increased sIgM levels in WEHI-231 cells. We found 2465 differentially expressed genes in BP7-treated WEHI-231 cells compared with control cells by gene microarray analysis. Furthermore, the differentially expressed genes were involved in 13 significantly enriched pathways in the BP7-treated WEHI-231 cells, of which ubiquitin-mediated proteolysis was the vital pathway. Ubiquitin-mediated processes have been reported to participate in MHCII complexes, which facilitate the B cell response and selection in germinal centres [33] and B cell receptor signalling [34]. These results proved the substantial mechanism underlying the effects of BP7 on the gene expression profile and signalling pathways in WEHI-231 cells, suggesting that BP7 might play a vital role in immature B cell development.

To investigate the molecular basis of the function of BP7 in immature B cells, we further analysed enriched GO terms with *p* values within TOP30 in WEHI-231 cells after BP7 treatment. It was observed that among the GO terms with *p* values within TOP30, 73.33% of the GO terms belonged to biological processes and 13.33% belonged to molecular functions in the BP7-treated WEHI-231 cells. In this paper, we further investigated the molecular mechanism underlying the role of BP7 in immune induction in immature mouse B cells. The immune-related GO terms and differentially expressed genes involved in BP7-treated WEHI-231 cells consisted of negative regulation of T cell and lymphocyte proliferation, T helper 2 cell differentiation, antigen processing and presentation, MHC class II and MHC class I biosynthetic processes, B cell activation and B cell-mediated immunity, and cytokine activity and secretion. The activation and proliferation of lymphocytes, including T and B cells, play vital roles in adaptive immune responses during vaccination [35, 36]. MHC class II and MHC class I are both principal components in antigen processing and presentation pathways [37]. These results suggested that BP7 might participate in various immune-related biological processes, resulting in multifunctional inducible functions in both humoural and cellular immune responses.

Autophagy is involved in various immune-related biological processes, including the development of B and T cells, antigen presentation by B cells, and the survival of memory lymphocytes and antibody-producing plasma cells [38–40]. In this study, BP7 regulated various differentially expressed genes involved in the autophagy pathway and autophagy-related biological processes, increased intracellular autophagosome formation and stimulated the expression of the LC3 protein in WEHI-231 cells. Additionally, we found that BP7 promoted AMPK and ULK1 phosphorylation and induced BCL-2 expression in WEHI-231 cells. An interaction between AMPK and the PS domain of ULK1 is required for ULK1-mediated autophagy [41]. Bcl-2 family members are dual regulators of apoptosis and autophagy [42]. These results suggested that BP7 could induce autophagy in immature B cells. However, the function and mechanism of autophagy induced by BP7 stimulation in immature B cell development and differentiation needs to be further explored in future work.

The regulatory function of active peptides in animal models is an important reference for clinical applications. In this study, we observed that BP7 induced strong antibody and cytokine responses in chicken immunization experiments. These results proved that BP7 could act as an immune-enhancing agent for an AIV vaccine, providing an important reference for clinical disease prevention and vaccine improvement.

In brief, the BF plays vital roles in B cell differentiation and antibody production, in which bursal-derived peptides may be involved in various functional processes and signalling activation during B cell development. In this study, a new regulatory heptapeptide, BP7, was isolated from the BF and induced strong antibody responses and cell-mediated immune responses specific to an AIV antigen in mouse immunization experiments and significantly increased sIgM levels in immature mouse B cells. Furthermore, BP7 regulated the expression of various genes involved in signalling pathways and immune-related biological processes and regulated autophagy through AMPK-ULK1 phosphorylation in immature B cells. Finally, BP7 promoted strong immune responses to the AIV antigen in chicken vaccination. These results provide novel insights into the molecular mechanism involving this bursal peptide in immune functions and B cell development.

## Supplementary information

**Additional file 1. MS/MS analysis of the amino acid sequence of BP7.** A) The parameters for MS/MS analysis of BP7. B) The MS/MS analysis information for BP7. C) Composition of seven amino acids.

**Additional file 2. Quantitative real-time PCR primers for the six selected genes used in this study.**

**Additional file 3. Alignments between BP7 and homologous proteins.**

**Additional file 4. Gene expression profiles of WEHI-231 cells treated with BP7.**

**Additional file 5. Enriched pathways and differentially expressed genes in WEHI-231 cells treated with BP7.**

**Additional file 6. Immune-related function terms and differentially expressed genes in WEHI-231 cells.**

## Data Availability

The datasets described and/or analysed during the current study are available from the corresponding author on reasonable request.

## References

[1] Kaiser P, Rothwell L, Galyov EE, Barrow PA, Burnside J, Wigley P (2000). Differential cytokine expression in avian cells response to invasion by *Salmonella typhimurium*, *Salmonella enteritidis* and *Salmonella gallinarium*. Microbiology.

[2] Natale G, Bocci G, Ribatti D (2017). Scholars and scientists in the history of the lymphatic system. J Anat.

[3] Gitlin AD, Nussenzweig MC (2015). Immunology: fifty years of B lymphocytes. Nature.

[4] Ratcliffe MJ (2006). Antibodies, immunoglobulin genes and the bursa of Fabricius in chicken B cell development. Dev Comp Immunol.

[5] Ratcliffe MJH, Härtle S, Schat KA, Kaspars B, Kaiser P (2014). B cells, the bursa of Fabricius and the generation of antibody repertoires. Avian immunology.

[6] Liu XD, Zhang F, Shan H, Wang SB, Chen PY (2016). mRNA expression in different developmental stages of the chicken bursa of Fabricius. Poult Sci.

[7] Audhya T, Kroon D, Heavner G, Viamontes G, Goldstein G (1986). Tripeptide structure of bursin, a selective B-cell-differentiating hormone of the bursa of fabricius. Science.

[8] Brand A, Gilmour DG, Goldstein G (1976). Lymphocyte-differentiating hormone of bursa of fabricius. Science.

[9] Baba T, Kita M (1977). Effect of extracts of the bursa of Fabricius on IgG antibody production in hormonally bursectomized chicken. Immunology.

[10] Liu XD, Zhang FB, Shan H, Chen PY (2015). The potential mechanism of bursal-derived BP8 on B cell developments. Biotechnol Lett.

[11] Feng X, Cao R, Zhou B, Liu Q, Liu K, Liu X, Zhang Y, Gu J, Miao D, Chen P (2013). The potential mechanism of Bursal-derived BPP-II on the antibody production and avian pre-B cell. Vaccine.

[12] Liu XD, Zhou B, Feng XL, Cao RB, Chen PY (2014). BP8, a novel peptide from avian immune system, modulates B cell developments. Amino Acids.

[13] Liu XD, Zhou B, Cao RB, Feng XL, Ma ZY, Chen PY (2014). BP5 regulated B cell development promoting anti-oxidant defence. Amino Acids.

[14] Feng XL, Liu QT, Cao RB, Zhou B, Ma ZY, Deng WL, Wei JC, Qiu YF, Wang FQ, Gu JY, Wang FJ, Zheng QS, Ishag H, Chen PY (2012). Identification and characterization of novel immunomodulatory bursal-derived pentapeptide-II (BPP-II). J Biol Chem.

[15] International Chicken Genome Sequencing Consortium (2004). Sequence and comparative analysis of the chicken genome provide unique perspectives on vertebrate evolution. Nature.

[16] Deng WL, Guan CY, Liu K, Zhang XM, Feng XL, Zhou B, Su XD, Chen PY (2014). Fine mapping of linear epitope on EDIII of Japanese encephalitis virus using a novel neutralizing monoclonal antibody. Virus Res.

[17] Feng XL, Liu QT, Cao RB, Zhou B, Zhang YP, Liu K, Liu XD, Wei JC, Li XF, Chen PY (2012). Characterization and immunomodulatory function comparison of various bursal-derived peptides isolated from the humoral central immune organ. Peptides.

[18] Marshall DR, Olivas E, Andreansky S, La Gruta NL, Neale GA, Gutierrez A, Wichlan DG, Wingo S, Cheng C, Doherty PC, Turner SJ (2005). Effector CD8+ T cells recovered from an influenza pneumonia differentiate to a state of focused gene expression. Proc Natl Acad Sci U S A.

[19] Feng XL, Zong MM, Zhou GF, Zheng Y, Yu YN, Cao RB, Chen PY, Yang M (2019). The functions and mechanism of a new oligopeptide BP9 from avian bursa on antibody responses, immature B cell, and autophagy. J Immunol Res.

[20] Looney BM, Xia CQ, Concannon P, Ostrov DA, Clare-Salzler MJ (2015). Effects of type 1 diabetes associated IFIH1 polymorphisms on MDA5 function and expression. Curr Diabetes Rep.

[21] Della Mina E, Rodero MP, Crow YJ (2017). Polymorphisms in IFIH1: the good and the bad. Nat Immunol.

[22] Qiu L, Zhou Y, Yu Q, Zheng S, Wang Z, Huang Q (2018). Elevated levels of follicular T helper cells and their association with therapeutic effects in patients with chronic lymphocytic leukaemia. Immunol Lett.

[23] Lehmann-Horn K, Wang SZ, Sagan SA, Zamvil SS, von Büdingen HC (2016). B cell repertoire expansion occurs in meningeal ectopic lymphoid tissue. JCI Insight.

[24] Sunay MME, Martins KAO, Steffens JT, Gregory M, Vantongeren SA, Van Hoeven N, Garnes PG, Bavari S (2019). Glucopyranosyl lipid adjuvant enhances immune response to Ebola virus-like particle vaccine in mice. Vaccine.

[25] Schneider-Ohrum K, Bennett AS, Rajani GM, Hostetler L, Maynard SK, Lazzaro M, Cheng LI, O’Day T, Cayatte C (2019). CD4+ T cells drive lung disease enhancement induced by immunization with suboptimal doses of RSV fusion protein in the mouse model. J Virol.

[26] Walker KW, Salimi-Moosavi H, Arnold GE, Chen Q, Soto M, Jacobsen FW, Hui J (2019). Pharmacokinetic comparison of a diverse panel of non-targeting human antibodies as matched IgG1 and IgG2 isotypes in rodents and non-human primates. PLoS One.

[27] Dobaño C, Santano R, Vidal M, Jiménez A, Jairoce C, Ubillos I, Dosoo D, Aguilar R, Williams NA, Díez-Padrisa N, Ayestaran A, Valim C, Asante KP, Owusu-Agyei S, Lanar D, Chauhan V, Chitnis C, Dutta S, Angov E, Gamain B, Coppel RL, Beeson JG, Reiling L, Gaur D, Cavanagh D, Gyan B, Nhabomba AJ, Campo JJ, Moncunill G (2019). Differential patterns of IgG subclass responses to *Plasmodium falciparum* antigens in relation to malaria protection and RTS, S vaccination. Front Immunol.

[28] Moran TM, Park H, Fernandez-Sesma A, Schulman JL (1999). Th2 responses to inactivated influenza virus can Be converted to Th1 responses and facilitate recovery from heterosubtypic virus infection. J Infect Dis.

[29] Proietti E, Bracci L, Puzelli S, Di Pucchio T, Sestili P, De Vincenzi E, Venditti M, Capone I, Seif I, De Maeyer E, Tough D, Donatelli I, Belardelli F (2002). Type I IFN as a natural adjuvant for a protective immune response: lessons from the influenza vaccine model. J Immunol.

[30] Hsieh CS, Heimberger AB, Gold JS, O’Garra A, Murphy KM (1992). Differential regulation of T helper phenotype development by interleukins 4 and 10 in an alpha beta T-cell-receptor transgenic system. Proc Natl Acad Sci U S A.

[31] Farrar MA, Schreiber RD (1993). The molecular cell biology of interferon-gamma and its receptor. Annu Rev Immunol.

[32] Tanabe K, Inui S (2015). Dominant negative form of alpha4 inhibits the BCR crosslinking-induced phosphorylation of Bcl-xL and apoptosis in an immature B cell line WEHI-231. Biomed Res.

[33] Bannard O, McGowan SJ, Ersching J, Ishido S, Victora GD, Shin JS, Cyster JG (2016). Ubiquitin-mediated fluctuations in MHC class II facilitate efficient germinal center B cell responses. J Exp Med.

[34] Wang X, Li JP, Kuo HK, Chiu LL, Dement GA, Lan JL, Chen DY, Yang CY, Hu H, Tan TH (2012). Down-regulation of B cell receptor signaling by hematopoietic progenitor kinase 1 (HPK1)-mediated phosphorylation and ubiquitination of activated B cell linker protein (BLNK). J Biol Chem.

[35] Lund FE, Randall TD (2010). Effector and regulatory B cells: modulators of CD4+ T cell immunity. Nat Rev Immunol.

[36] Li MO, Rudensky AY (2016). T cell receptor signalling in the control of regulatory T cell differentiation and function. Nat Rev Immunol.

[37] Neefjes J, Jongsma ML, Paul P, Bakke O (2011). Towards a systems understanding of MHC class I and MHC class II antigen presentation. Nat Rev Immunol.

[38] Vyas JM, Van der Veen AG, Ploegh HL (2008). The known unknowns of antigen processing and presentation. Nat Rev Immunol.

[39] Arnold J, Murera D, Arbogast F, Muller S, Gros F (2016). Autophagy in T and B cell homeostasis: recycling for sustainable growth. Med Sci.

[40] Fribourg M, Ni J, Nina Papavasiliou F, Yue Z, Heeger PS, Leventhal JS (2018). Allospecific memory B cell responses are dependent on autophagy. Am J Transplant.

[41] Lee JW, Park S, Takahashi Y, Wang HG (2010). The association of AMPK with ULK1 regulates autophagy. PLoS One.

[42] Levine B, Sinha SC, Kroemer G (2008). Bcl-2 family members: dual regulators of apoptosis and autophagy. Autophagy.

