# Correction to: The immunomodulatory functions and molecular mechanism of a new bursal heptapeptide (BP7) in immune responses and immature B cells

**DOI:** 10.1186/s13567-020-00884-9

**Published:** 2021-02-16

**Authors:** Xiu Li Feng, Yang Zheng, Man Man Zong, Shan Shan Hao, Guang Fang Zhou, Rui Bing Cao, Pu Yan Chen, Qing Tao Liu

**Affiliations:** 1 Key Laboratory of Animal Microbiology of China’s Ministry of Agriculture, College of Veterinary Medicine, Nanjing Agricultural University, Nanjing, 210095 China; 2 MOE Joint International Research Laboratory of Animal Health and Food Safety, College of Veterinary Medicine, Nanjing Agricultural University, Nanjing, 210095 China; 3 Institute of Veterinary Medicine, Jiangsu Academy of Agricultural Sciences, Nanjing, 210014 China

## Correction to: Vet Res (2019) 50:64 10.1186/s13567-019-0682-7

Following publication of the original article [1], the authors identified an error in the author name of Qing Tao Liu. The given names were erroneously transposed.

The incorrect author name is: Tao Qing Liu.

The correct author name is: Qing Tao Liu.

The author group has been updated above and the original article [1] has been corrected.

## References

[1] Feng XL, Zheng Y, Zong MM, Hao SS, Zhou GF, Cao RB, Chen PY, Liu QT (2019). The immunomodulatory functions and molecular mechanism of a new bursal heptapeptide (BP7) in immune responses and immature B cells. Vet Res.

