# Supplementary figures and images for: The immunomodulatory functions and molecular mechanism of a new bursal heptapeptide (BP7) in immune responses and immature B cells
Source: Vet Res. 2019 Sep 18;50:64. doi: 10.1186/s13567-019-0682-7 (PMC6749628; doi:10.1186/s13567-019-0682-7)

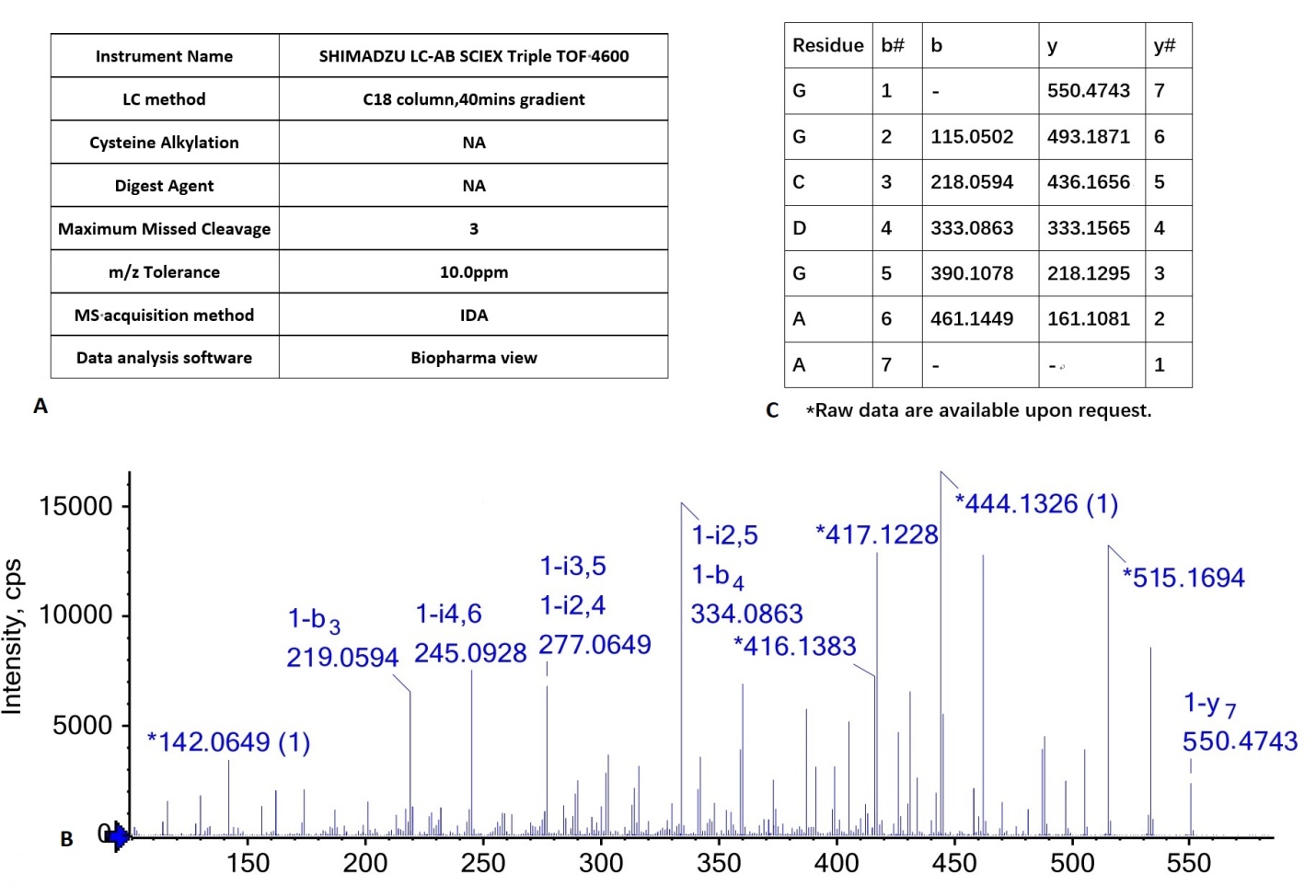

Supplement: Supplementary file 1 — Additional file 1. MS/MS analysis of the amino acid sequence of BP7. A) The parameters for MS/MS analysis of BP7. B) The MS/MS analysis information for BP7. C) Composition of seven amino acids. [file 13567_2019_682_MOESM1_ESM.docx]
