# Supplementary material for: The immunomodulatory functions and molecular mechanism of a new bursal heptapeptide (BP7) in immune responses and immature B cells
Source: Vet Res. 2019 Sep 18;50:64. doi: 10.1186/s13567-019-0682-7 (PMC6749628; doi:10.1186/s13567-019-0682-7)
Supplement: Supplementary file 2 — Additional file 2. Quantitative real-time PCR primers for the six selected genes used in this study. [file 13567_2019_682_MOESM2_ESM.docx]

| Genes | Access Number | Primer sequence | |
| --- | --- | --- | --- |
| Sos1 | NM_009231 | Forward | cacagttgagtggcacataagc |
|  |  | Reverse | tttctcaaaccacaaagtgagg |
| Atg14 | NM_172599 | Forward | atcatattcccaatcgacgaag |
|  |  | Reverse | gtggtcatcacagacccatctt |
| Atg12 | NM_026217 | Forward | cattgtgatccatacctgctgt |
|  |  | Reverse | gagaagtgagggttgggagat |
| Pias3 | NM_018812 | Forward | cctggattactcttgcaccttt |
|  |  | Reverse | acgctcacagaacattcacaac |
| Ube2b | NM_009458 | Forward | cacaccagcctcctagagatgt |
|  |  | Reverse | catgcagtaaccactgaaccat |
| Ifnb1 | NM_010510 | Forward | tattactggagggtgcaaaggt |
|  |  | Reverse | ttcactaccagtcccagagtcc |
