# Supplementary material for: The immunomodulatory functions and molecular mechanism of a new bursal heptapeptide (BP7) in immune responses and immature B cells
Source: Vet Res. 2019 Sep 18;50:64. doi: 10.1186/s13567-019-0682-7 (PMC6749628; doi:10.1186/s13567-019-0682-7)
Supplement: Supplementary file 3 — Additional file 3. Alignments between BP7 and homologous proteins. [file 13567_2019_682_MOESM3_ESM.docx]

| Species | Homologous proteins | Alignments |
| --- | --- | --- |
| G. Gallus | BP7 | 1 GGCDGAA 7  186 GGCDLAA 192 |
|  | Interferon-induced helicase C domain-containing protein 1 |  |
|  | BP7 | 1 GGCDGA 6  109 GGCHGA 114 |
|  | Immunoglobulin heavy chain variable region (IGHV) |  |
| M.musculus | BP7 | 1 GGCDGAA 7  12 GGCDVAA 18 |
|  | diacylglycerol kinase zeta isoform X6 |  |
|  | BP7 | 1 GGCDGA 6  141 GGCDGA 146 |
|  | transducin (beta)-like 3, isoform CRA_b |  |

Note: G. Gallus, Gallus. gallus; M.musculus, Mus musculus.
