# Supplementary material for: The immunomodulatory functions and molecular mechanism of a new bursal heptapeptide (BP7) in immune responses and immature B cells
Source: Vet Res. 2019 Sep 18;50:64. doi: 10.1186/s13567-019-0682-7 (PMC6749628; doi:10.1186/s13567-019-0682-7)
Supplement: Supplementary file 5 — Additional file 5. Enriched pathways and differentially expressed genes in WEHI-231 cells treated with BP7. [file 13567_2019_682_MOESM5_ESM.docx]

|  | Term | Database | ID | Corrected *P*-Value | Up regulated genes | Down regulated genes |
| --- | --- | --- | --- | --- | --- | --- |
| biosynthesis and metabolism | Glycosphingolipid biosynthesis - lacto and neolacto series | KEGG PATHWAY | mmu00601 | 0.9493647 | B3galt2, Gcnt2 | Sec1, B3gnt4, B4galt3 |
|  | Fatty acid biosynthesis | KEGG PATHWAY | mmu00061 | 0.9493647 | Acsl5, Acacb | Acsl1 |
|  | Ribosome biogenesis in eukaryotes | KEGG PATHWAY | mmu03008 | 0.9493647 | Pop4, Gar1, Rrp7a, Pop1, Rpp25, Riok1 | Drosha, Csnk2b, Csnk2a1, Pop5 |
|  | Choline metabolism in cancer | KEGG PATHWAY | mmu05231 | 0.9493647 | Pla2g4c, Ppap2c, Sos1 | Slc44a3, Pik3ca, Fos, Hif1a, Chka, Slc44a4, Pdgfb |
|  | Ubiquitin mediated proteolysis | KEGG PATHWAY | mmu04120 | 0.9493647 | Ube2a, Klhl9, Nedd4l, Nhlrc1, Fbxw8 | Pias3, Fancl, Ube2b, Ube2o, Sae1, Ube2d2b, Ube4b, Ube2l3 |
| signal pathway | RIG-I-like receptor signaling pathway | KEGG PATHWAY | mmu04622 | 0.9493647 | Atg12, D1Pas1 | Ddx3x, Tank, Ifna7, Ifnb1, Ifna2, Map3k7 |
|  | Thyroid hormone signaling pathway | KEGG PATHWAY | mmu04919 | 0.9493647 | Myh6, Hdac1 | Plcb2, Ncor1, Pik3ca, Kat2a, Hif1a, Foxo1, Med24, Med30, Bmp4, Med16 |
|  | Fanconi anemia pathway | KEGG PATHWAY | mmu03460 | 0.9493647 | Fance, Fancb, Ube2t | Fan1, Fancl, Brip1, Atrip, Rpa1 |
|  | HIF-1 signaling pathway | KEGG PATHWAY | mmu04066 | 0.9493647 | Pgk1, Gapdh, Egln3, Flt1, Il6ra, Ifng, Eif4e | Pik3ca, Hif1a, 1300017J02Rik, Hk1, 15275 |
|  | mTOR signaling pathway | KEGG PATHWAY | mmu04150 | 0.9493647 | Stk11, Pten, Mlst8, Eif4e | Rragc, Hif1a, Pik3ca |
| other | Basal transcription factors | KEGG PATHWAY | mmu03022 | 0.9493647 | Taf7l, Taf5l, Taf5, 4933416C03Rik | Gtf2h2, Ercc3, Taf1, Gtf2i |
|  | Regulation of autophagy | KEGG PATHWAY | mmu04140 | 0.9493647 | Atg14, Atg12, Ifng, Atg13 | Ifna7, Ifna2, Atg16l2 |
|  | RNA transport | KEGG PATHWAY | mmu03013 | 0.9493647 | Pop4, Snupn, Eef1a2, Pop1, Gm9839, Rpp25, Upf3b, Eif4e | Nup210, Senp2, Ncbp1, Tacc3, Gm5415, Rangap1, Pop5, Ncbp2 |
