# Supplementary material for: The immunomodulatory functions and molecular mechanism of a new bursal heptapeptide (BP7) in immune responses and immature B cells
Source: Vet Res. 2019 Sep 18;50:64. doi: 10.1186/s13567-019-0682-7 (PMC6749628; doi:10.1186/s13567-019-0682-7)
Supplement: Supplementary file 6 — Additional file 6. Immune-related function terms and differentially expressed genes in WEHI-231 cells. [file 13567_2019_682_MOESM6_ESM.docx]

|  | #Term | ID | Corrected *P*-Value | Up-regulated genes | Down-regulated genes |
| --- | --- | --- | --- | --- | --- |
| T cell | negative regulation of T cell proliferation | GO:0042130 | 0.9493647 | Zc3h12d, Ctla4, Spn, Il2ra, Pde5a, Dlg1 | Bmp4, March7 |
|  | negative regulation of T cell activation | GO:0050868 | 0.9493647 | Zc3h12d, Ctla4, Spn, Il2ra, Pde5a, Dlg1 | Ifnb1, Bmp4, March7 |
|  | negative regulation of lymphocyte proliferation | GO:0050672 | 0.9493647 | Zc3h12d, Ctla4, Spn, Il2ra, Pde5a, Dlg1 | Bmp4, March7 |
| antigen processing and presentation | antigen processing and presentation | GO:0019882 | 0.9493647 | Tap2, Rab4a, Raet1a, Rab32, Wash, Ifng | Cd1d2, Rab8b |
| Th2 | T-helper 2 cell differentiation | GO:0045064 | 0.9493647 | Bcl3, Gata3 | Rara |
| MHC II and I | regulation of MHC class II biosynthetic process | GO:0045346 | 0.9493647 | Ifng, Ciita | Nfx1 |
|  | MHC class II biosynthetic process | GO:0045342 | 0.9493647 | Ifng, Ciita | Nfx1 |
|  | regulation of MHC class I biosynthetic process | GO:0045343 | 0.9493647 | Nlrp12, Ciita | Ifnb1 |
|  | MHC class I biosynthetic process | GO:0045341 | 0.9493647 | Nlrp12, Ciita | Ifnb1 |
| Interleukin | interleukin-1 binding | GO:0019966 | 0.9493647 | Il1rn,Hax1 |  |
|  | positive regulation of interleukin-5 production | GO:0032754 | 0.9493647 | Gata3 | Rara |
| autophagy | autophagy | GO:0006914 | 0.9493647 | Stk11, Wash, Nhlrc1, Fundc1, Cln3, Rnf41, Atg14, Atg12, Sirt2, Vmp1, Atg13 | Tecpr1, Rragc, Hif1a, Usp13, Bnip1, Trim13, Foxo1, Atg16l2, 1700021K19Rik |
|  | regulation of autophagy | GO:0010506 | 0.9493647 | Wash, Cln3, Atg14, Sirt2, Vmp1, Atg13 | Rragc, Usp13, Hif1a, Trim13, Foxo1, 1700021K19Rik |
